# Role of TGF-β in Skin Chronic Wounds: A Keratinocyte Perspective

**DOI:** 10.3390/cells9020306

**Published:** 2020-01-28

**Authors:** Sergio Liarte, Ángel Bernabé-García, Francisco J. Nicolás

**Affiliations:** Laboratorio de Regeneración, Oncología Molecular y TGF-β, IMIB-Arrixaca, El Palmar, 30120 Murcia, Spain; a.bernabegarcia@gmail.com

**Keywords:** TGF-β, keratinocytes, chronic wounds, wound healing

## Abstract

Chronic wounds are characterized for their incapacity to heal within an expected time frame. Potential mechanisms driving this impairment are poorly understood and current hypotheses point to the development of an unbalanced milieu of growth factor and cytokines. Among them, TGF-β is considered to promote the broadest spectrum of effects. Although it is known to contribute to healthy skin homeostasis, the highly context-dependent nature of TGF-β signaling restricts the understanding of its roles in healing and wound chronification. Historically, low TGF-β levels have been suggested as a pattern in chronic wounds. However, a revision of the available evidence in humans indicates that this could constitute a questionable argument. Thus, in chronic wounds, divergences regarding skin tissue compartments seem to be characterized by elevated TGF-β levels only in the epidermis. Understanding how this aspect affects keratinocyte activities and their capacity to re-epithelialize might offer an opportunity to gain comprehensive knowledge of the involvement of TGF-β in chronic wounds. In this review, we compile existing evidence on the roles played by TGF-β during skin wound healing, with special emphasis on keratinocyte responses. Current limitations and future perspectives of TGF-β research in chronic wounds are discussed.

## 1. Introduction

Chronic wounds are characterized by being unable to heal in an expected time frame. This is often related to defects in keratinocyte ability to epithelialize over recovered dermal tissue. In healthy unaltered epidermis, keratinocytes in the basal layer proliferate slowly to contribute to tissue homeostasis. They later differentiate while progressing into suprabasal layers until becoming mere keratin scales in the stratum corneum. Yet, since they are the main constituent of the exposed organism physical barriers, keratinocytes in the epidermis seem to be prepared to quickly respond to environmental injury. Across strata, living keratinocytes express cell mediators and showcase receptors ready to signal in case of harm. Amid other effects, signaling through these factors triggers keratinocyte activation in order to contribute to tissue repair. Upon the eventual re-establishment of epithelial continuity, keratinocyte phenotype would revert to the initial state, thus completing the keratinocyte activation cycle [1]. Alterations of this process could result in unsuccessful wound closure. This situation commonly associates with persistent inflammation, which perpetuates a non-healing state, thus promoting ulcer chronicity and frequent relapse along with the development of comorbid complications (i.e., infections) [2]. Although the etiologies leading to the development of chronic wounds are fairly well understood, whether in venous stasis ulcers, chronic pressure ulcers, diabetic ulcers, or massive traumatic wounds, the molecular events leading to failure of keratinocytes to help closure are, generally, poorly understood.

Throughout the diversity of growth factors and cytokines known to influence wound healing, TGF-β is considered to promote the broadest spectrum of effects [3,4]. Initially conceived as a rather simple linear non-amplified signaling pathway, accumulated research on TGF-β has demonstrated otherwise, with new implications still emerging. In developing embryos, members of the TGF-β superfamily, including bone morphogenetic proteins (BMPs), growth and differentiation factors (GDFs), activins, and TGF-βs, mediate key intercellular communications necessary for adequate tissue development and for the arrangement of the overall body plan. Not by accident, the origins of the superfamily date back to non-bilaterian animal forms of life. Being one of the youngest within this superfamily, the rise of the TGF-β family itself is linked to the emergence of the anteroposterior/ventral–dorsal bilateral symmetry which defines most metazoans today, which originated between 635 and 541 million years ago during the Ediacaran Period [5]. Consequently, TGF-β signaling had quite enough time to evolve and finely specialize as well as to relate to other pathways. In adult mammals, responses to its signaling range from damaged tissue repair, extracellular matrix (ECM) maintenance, epithelial and endothelial cell growth, and differentiation to the regulation of immune responses [6]. This constitutes an evolutionary wonder which introduces great difficulty in understanding some cellular behaviors at the molecular level. In fact, that would be the case for TGF-β isoforms role in skin chronic wounds which, although regarded as relevant, remains elusive and is yet to be completely addressed [7]. In this paper, we aim to recapitulate useful evidence relevant to the issue, discussing current limitations and future perspectives in the research of TGF-β’s role in chronic wounds with a special focus on keratinocytes.

## 2. TGF-β Signaling: A Context Dependent Mechanism

The TGF-β family comprises *TGFB1*, *TGFB2*, and *TGFB3*. All three genes are highly conserved across species and humans, in which their products share strong sequence similarity and also display nearly identical three-dimensional structures [8,9]. They signal through the same ubiquitously expressed transmembrane receptors, generally referred to as TβRI and TβRII, which develop a similar affinity for isoforms TGF-β1 and TGF-β3, whereas only TβRII binds with less intensity to TGF-β2 [10,11]. Thus far, the main element discriminating physiological roles for the three TGF-β isoforms might be differences in their spatial and temporal expression patterns. However, molecular recognition of TGF-β is not achieved via simple ligand–receptor interaction, but through a network of interactions deeply affecting the final outcome. For starters, TGF-β is usually regarded as a homodimer, yet heterodimeric configurations showing variable potency and binding affinity with TβRs have been also reported both in vivo and in vitro [12,13]. Once secreted, TGF-β can remain in a latent state for some time, allowing for stock build-up in the extracellular matrix (ECM). This arises from the presence of the latency associated peptide prodomain (LAP). Indeed, TGF-β is translated into single polypeptide chains containing both a monomer and its corresponding LAP whose maturation through the trans-Golgi network involves the formation of disulfide bonds resulting in dimer stabilization. The following proteolytic cleavage splits polypeptide chains; however, the association between LAP and TGF-β remains stable through non-covalent interaction. LAP removal constitutes a critical regulatory event for TGF-β activity achieved through diverse mechanisms, which range from conformational changes promoted by interaction with integrins to enzymatic digestion including matrix metalloproteinases [14,15]. In that sense, and in contrast to TGF-β isoforms, LAPs show four times greater sequence divergence, allowing for diversification of the activation dynamics [5]. Additionally, modulators already present on secretion, such as latent TGF-β binding proteins (LTBPs), or in the ECM, such as decorin, biglycan, or fibromodulin, bind and delay TGF-β activation [16,17]. Moreover, co-receptor molecules with the ability to modulate and which bind to TβRs have been described [18]. Furthermore, downstream to TβRs activation, intracellular transduction of the signal shows, again, possibilities for modulation by means of post-translational modifications [19,20] or the regulation of protein levels of the factors participating on either Smad-reliant canonical signaling [21] or MAP-kinase-dependent non-canonical signaling [22]. In that sense, the aspects of TGF-β signaling intracellular transduction and the crosstalk with other pathways have been the subject of extensive reviews [23,24,25,26]. In the end, the ability of TGF-β stimulation to promote long-term modulation of gene expression is affected by concomitant circumstances, for instance, the epigenetic status or the microRNA profile of the targeted cell [27,28], among others. Altogether, the sum of mechanisms involved in TGF-β signaling implies countless possibilities for modulation at any level, making the final outcome highly dependent on cell type and context. This fact encourages the shift of the paradigm for TGF-β pathway from linear non-amplified signaling cascades to complex signaling networks, better explaining the variety of functional responses to TGF-β isoforms [5].

## 3. TGF-β Expression in Healthy and Healing Epidermis

In the skin, TGF-β isoforms are differentially expressed by nearly all classes of its constituent cells. For the case of healthy human epidermis, some TGF-β1 but mostly TGF-β3 expression has been described at the basal cellular layer [29,30]. This has been suggested to be constitutive and necessary for epithelial homeostasis [31,32,33]. In contrast, for the process of wound healing, most cells involved develop distinct spatial and temporal expression patterns of TGF-β [29]. Upon skin injury, TGF-β regional induction in the wound has been found to result in a double-peak availability pattern [34,35,36]. That pattern would emerge from the initial quick abundant platelet release, later building up on the aggregated contributions over time from endothelial cells, monocytes, fibroblast, and keratinocytes [37]. Specifically regarding the epidermis, it is acknowledged that TGF-β expression levels surge across strata during the course of acute wound healing; however, there is no clear description of the patterns for each TGF-β isoform [3]. Crosstalk with concurrent signaling might also affect the definite cellular responses characteristic of dermal regeneration, angiogenesis, and re-epithelization (reviewed in [38,39]). In this regard, it should be noted that growth factors like EGF, PDEGF, HIF, VEGF, and other cytokines expressed by the cells which constitute each tissue layer of the skin are known to affect responses at neighboring tissue compartments [40]. As a pertinent example of the previously mentioned crosstalk between tissue compartments, TGF-β produced by keratinocytes during acute wound healing is believed to induce the suppression of TGF-β release by fibroblasts [41]. Interestingly, that crosstalk has been described as necessary to revert keratinocytes to a basal state and complete the activation cycle [1].

## 4. Paradoxical Role of TGF-β in Inflammation

Within the context of skin, paradoxical effects have been attributed to TGF-β regarding its involvement in inflammation [42]. Early studies ascribed anti-inflammatory activity to TGF-β. This was based on the observation that TGF-β1 null mice died of excessive inflammation affecting several vital organs, including the heart and lungs, within days after birth [43,44]. Yet, pro-inflammatory effects have also been described, with TGF-β1 being accepted as a double-edged sword in this case. This double nature has been related to distinct actions over the innate and the adaptive immune responses. TGF-β1 is known to be a potent chemoattractant for endothelial cells and fibroblasts as well as for innate immune cells, such as neutrophils and monocytes [33]. Besides, it can trigger the release by all these cells of early-response pro-inflammatory cytokines, including interleukins (IL) IL-1b, IL-6, and TNF-α, also promoting macrophage differentiation (reviewed in [3]). Interestingly, at the same time, TGF-β1 is known to inhibit IL-2 production and to hinder naïve T-cell activation and differentiation in effector phenotypes [45]. As such, the described molecular milieu would be paramount in consolidating the inflammatory phase of wound healing and establishing a proportionate immune response. However, TGF-β activity has also been found to promote Th17 cell activation and differentiation [46]. Curiously, with regard to skin disorders and increased TGF-β expression, some studies suggest that pro-inflammatory effects are predominant [36,47].

Considering the above, continued or aberrant integration of these signaling mechanisms might constitute an original component for the persistent inflammation state found in chronic wounds. This notion would be supported by increased leukocyte counts found in TGF-β1 transgenic mice skin [48,49,50] in contrast with the absence of inflammatory processes in the skin of TGF-β1 knockouts [43,44]. Interestingly, this ability of TGF-β to affect inflammation appears to be dependent on Smad3 canonical signaling, as Smad3 knockout mice show significantly reduced monocyte infiltration in wounds [51]. It has been proven that these mice also develop immune defects involving impaired neutrophil chemotactic responses and exacerbated T-cell activation in mucosae [52]. Altogether, different authors have prompted to interfere with these immunomodulatory effects for the conditions in which TGF-β imbalance is suspected to play a major role. Small molecule inhibitors, neutralizing antibodies, or exogenous TGF-β application have been tested as potential tools for the management of diseases whose inflammatory component allegedly responds either to increased TGF-β signaling, like psoriasis [53,54,55] or, on the contrary, to apparently reduced TGF-β levels, which is believed to be the case for chronic wounds [37,56]. Notwithstanding, reviews on the matter [33,57] have recorded little to no success for the case of chronic wounds and inflammation. These observations might raise the question of how persistent inflammatory infiltrates in chronic ulcers might relate to TGF-β levels. 

## 5. Animal Models: Relevant but Potentially Misleading Evidence

Right after its discovery, in the late eighties and the early nineties, studies focused on TGF-β1 effects in animal models. Several of those studies performed in rats, mice, or rabbits reported reduced TGF-β1 in relation to impaired wound healing [51,58,59,60,61,62,63]. This, along with the conception of the role of TGF-β in skin homeostasis and its induction during wound healing encouraged abundant research on the potential beneficial effects of the exogenous administration of TGF-β in complicated wounds. That notion, consolidated by reviews picking up on the idea of decreased TGF-β levels in human chronic wounds [37,56], has induced contemporary authors to contribute to the trend from time to time [64,65,66,67]. Despite the fact that contradictory evidence produced in animal models is rare [68,69,70], any positive outcome described in the limited attempts performed to assess the functionality of this strategy for chronic wounds in human patients is yet to be translated into efficacy (several reviews on the matter [3,71,72]). In relation to this, some have defended that the method of administration may hamper TGF-β capabilities. This has been argued especially for topical use, since the intense proteolytic activity prevailing in chronic wounds may result in quick degradation [3]. Besides, it is still questionable whether or not these animal models genuinely reproduce the characteristics of human chronic wounds. For instance, regarding the experimental settings applied, some might resemble conditions of acute healing rather than chronic wounds (for an extended review see [73]). Moreover, intrinsic biological differences may distort the applicability of some of the conclusions obtained on humans. Just anatomically, direct dissimilarities within the skin exist, such as the lack of eccrine glands of non-primate mammals or the relative scarcity of hair follicles in human skin. Far from anecdotic differences, those structures are believed to actively support wound healing by contributing stem cells to the re-epithelialization process [74]. In fact, recent studies, including a randomized controlled trial, suggest that introducing hair follicle grafts could accelerate venous chronic ulcer healing [75,76]. As a result of those histological divergences, differences are also expected at the molecular level. In this regard, re-epithelialization would be determined by the balance of a complex array of factors and conditions, including TGF-β, which may vary extensively in the wound site across species. In that sense, distinct patterns have been indicated for TGF-β expression in the skin. The presence of TGF-β2 in healthy human adult skin is characterized by moderate basal expression, which is slightly higher than for TGF-β1 or TGF-β3, however, its mRNA expression barely fluctuates during wound healing [16]. In mice skin, in contrast, moderate basal expression of TGF-β2 is reported as well, although it experiences a more evident induction during the course of wound healing [34]. It is worth noting that accumulated evidence both in mice and humans corroborate the proposal of a prominent role of TGF-β2 in the regulation of hair follicle physiology [77,78,79,80]. All this implies mechanistic differences between human and animal models in the regulation of the wound healing process. Those differences could result in dissimilar crosstalk between tissue compartments, or differences in the relative signaling by concurrent factors other than TGF-β isoforms, ultimately limiting the transapplicability of experimental conclusions.

## 6. TGF-β in Human Chronic Wounds

In spite of accumulated knowledge on the acute healing process, regarding human chronic skin lesions, few studies have provided meaningful evidence on TGF-β patterns according to tissue compartments. Interestingly, regardless of the type of ulcer, original research articles on this issue coincide in describing a noticeable lack of expression for all the TGF-β isoforms in the human chronic wound bed. These observations rely on results obtained by means of in situ hybridization, immunofluorescence, and immunohistochemistry techniques, complemented with PCR in some cases [30,81,82]. As mentioned before, this notion of reduced TGF-β levels in chronic wounds has prevailed in literature. Yet, although the dermal component constitutes the bulk in terms of the wound surface, these reduced levels would just circumscribe to it. In the same studies, observations regarding the epidermal compartment are contrasting. For decubitus sores, in situ hybridization studies have shown increased levels of both TGF-β1 and 3 in the hyperkeratotic epidermis surrounding the chronic ulcer, yet TGF-β2 expression was not detected [30]. Regarding venous stasis ulcers, immunofluorescent studies provide mixed evidence: some authors reported intense TGF-β1 expression in the epidermis next to the ulcer edge, along with reduced TGF-β3 and negligible TGF-β2 staining [81], in contrast with other authors, who reported moderate TGF-β2 and 3 levels with negligible TGF-β1 expression in biopsies that included epidermis next to the wound bed [82]. On the issue of diabetic foot ulcers, immunohistochemistry studies showed increased TGF-β3 levels across all epidermis layers. Interestingly, although the same studies showed modest TGF-β1 expression at the basal stratum in the ulcer edge, cells next to the wound edge clearly displayed intense staining [82]. Overall, this collective evidence suggests that augmented TGF-β levels are to be expected in the epidermis of human chronic wounds. Thus, while it is true that decreased TGF-β levels can be found in chronic wounds, a thorough review of aforementioned studies suggests that this statement would be rather inaccurate as it fails to convey the fact that high TGF-β levels are detected in the epidermis compartment. 

## 7. Characteristic TGF-β Responses of Skin Cells

For fibroblasts and endothelial cells, TGF-β is known to exert chemotactic and pro-mitotic activities essential for the constitution of functional granulation tissue during the inflammatory and proliferative phases of wound healing [83]. Later, TGF-β ability to promote fibronectin synthesis and collagen deposition by fibroblasts becomes crucial for proper ECM replacement during the remodeling phase [84,85]. Interestingly, in human fibroblasts, these responses have been reported to evolve as TGF-β concentration varies. Higher levels are considered better at promoting migration, in contrast with lower levels, which are better at inducing proliferation [86]. Moreover, while TGF-β levels remain far from those achieved through platelet degranulation, the second TGF-β peak, resulting from the contribution of cells constituent of the skin, has been reported to specifically contribute to wound contraction by prompting fibroblasts to differentiate into myofibroblasts [87,88]. Also, the combined effects of TGF-β3 and TGF-β1 have been found capable of fine-tuning collagen deposition [89,90], with relevant implications in the development of scarring and fibrosis [91]. It is worth mentioning that fibroblasts isolated from chronic venous ulcers have shown non-functional TGF-β signaling due to decreased TβRII expression. This results in a number of critical fibroblast abnormalities, including reduced proliferation and impaired migration [92].

Distinct and particular effects have seemingly been described for keratinocytes and TGF-β signaling. Varying TGF-β levels have been reported to be involved in the exchange of keratins typical of keratinocyte activation and their reversal to a basal phenotype [1]. TGF-β has also been suggested to promote keratinocyte migration through a mechanism involving integrin induction and Smad-dependent signaling [93]. Moreover, TGF-β has been suggested to mediate proliferative responses in activated keratinocytes. TGF-β inoculated in vitro is well known to induce robust G1 cell cycle arrest in epithelial cell lines [94], including keratinocytes [95]. That kind of response seems to be channeled through the Smads, since loss of or aberrant canonical signaling is a common feature of squamous cell carcinomas derived from epidermal keratinocytes [96]. Interestingly, studies performed on HaCaT cells describe how these keratinocytes experience TGF-β induction, mainly TFG-β1, during differentiation [97]. Moreover, they show TβR upregulation and sensitization to autocrine TGF-β in response to an initial TGF-β1 treatment [98]. Altogether, this is in line with the TGF-β double-peak dynamics mentioned earlier [35] as well as with the evidence indicating that after injury, initial keratinocyte proliferation is restricted to cells distal to the leading edge, with recruitment of those cells next to the wound edges not earlier than 2–3 days post-wounding [99,100]. 

## 8. Reaction to Exacerbated TGF-β Levels in Wound Healing

Based on what has been discussed above, it is plausible that excessive TGF-β levels occurring only in the epidermal layer at the edge of the ulcer might contribute to the chronification of the wound. This notion is supported by concurrent evidence obtained from animal and human study models. Constitutive and high expression of integrin αvβ6 has been linked to a chronic wound state [101]. Moreover, overexpression of this integrin has been associated with elevated TGF-β1 levels and spontaneous ulceration in mice [101]. Studies using transgenic mice expressing different TGF-β1 constructs reveal skin alterations similar to those found in chronic wounds. The first model ever described implemented a construct expressing constitutively active TGF-β1 under the control of the human keratin-1 promoter. These animals showed an altered phenotype characterized by restricted mobility and impaired breathing and died within a day after birth. Analysis of their skin revealed hyperkeratosis and decreased proliferation in the epidermis [102]. Later models expanded on this evidence as those mice reached adulthood. This was achieved by implementing complete TGF-β1 sequences along with either human keratin-5 (hK5) or keratin-14 (hK14) promoters, which restrict transgene expression to the basal keratinocyte layer [31]. These advanced models differed greatly in their phenotype, ranging from hK14 shabby aspect [49] to hK5 scaly and erythematous skin similar to psoriatic erythroderma [48]. Notably, while TGF-β1 levels in unwounded skin of hK14 mice were similar to TGF-β1 levels in wild type [49], persistent levels similar to those occurring in acute wounds were reported for the hK5 models [48,50]. At the microscope, hK5 mice showed altered epidermis with signs of acanthosis (hyperplastic epidermis), diminished granular layer, and thickened stratum corneum, in some cases also showing abnormal basal and follicular keratinocyte proliferation in the form of hyperplasia and hyperkeratosis [48,50]. Moreover, some of these models developed spontaneous ulcerations in friction areas [36]. Interestingly, regardless of the promoter used, all these mice models developed significant delay in full-thickness wound recovery in comparison with non-transgenic mice [36,49,65,103]. By contrast, results obtained from TGF-β1 knockout mice showed regular wound healing and, in some cases, accelerated recovery [69,104]. In sum, the evidence obtained in transgenic mice constitutively overexpressing TGF-β1 in keratinocytes supports the hypothesis that persistent TGF-β signaling might not benefit wound healing and might constitute a relevant element in the pathogenesis of chronic wounds. 

## 9. Epithelial to Mesenchymal Transition During Wound Healing and the Involvement of TGF-β

Though devoid of proliferation, keratinocytes at the edge of acute wounds experience changes similar to epithelial–mesenchymal transition (EMT). The concept of EMT implies a dramatic phenotypical switch providing epithelial cells with unusual capacities, like high mobility, the ability to surpass basement membranes, and resistance to apoptosis [105]. These are considered unequivocal traits of advanced states in tumor transformation and are necessary for the progression of epithelium-derived cancer in terms of invasiveness and metastatic potential [105]. Diverse molecular processes engage in this phenomenon and contribute to its development. These processes include signals from growth factor and cytokines, including EGF, FGF, HGF, KGF, and TGF-β, promoting the activation of transcription factors, reorganization of cell architecture, expression of specific surface proteins, production of ECM-degrading enzymes, and modulation of specific microRNAs [105]. Biased response to TGF-β is considered a major mechanism for EMT in cancer, this being the subject matter of numerous reviews [106,107]. Indeed, biased responses affect the expression of key cell adherence and migration markers, among other known effects [106,107,108,109,110]. These include Smad canonical signaling driving the expression of key transcription factors such as Slug or Snail [111], which mediate reduced E-cadherin detection and induction of mesenchymal markers such as vimentin, the two latter effects being recognized EMT hallmarks [112]. Concurrent signaling involving MAP-kinases has also been suggested to contribute to this phenomenon [113]. 

The transition phenomenon described above develops less intensely in skin wounds. In that context, keratinocytes are reported to experience temporal transdifferentiation involving cytoskeleton reorganization, loss of cell polarity, and partial dissociation of adhesion structures [114]. This is supposed to allow cells to minimize attachment to the basal membrane while elongating and acquiring motility, seeking to re-establish epithelial coherence [115]. While these transdifferentiation dynamics are fairly well understood, no consensus exists on how keratinocyte activities coordinate for re-epithelization. Several models have been proposed for the discussion on whether proliferation at the wound edge and migration occurs in the basal, suprabasal, or both layers [115]. Moreover, specifically for migration, the considerations in the available literature regarding keratinocyte transdifferentiation during wound healing are mostly based on what is known about TGF-β and EMT during development and cancer [38,114,116]. Interestingly, in light of the accumulated evidence, different review authors point to the existence of intermediate EMT states [117,118]. Though it is still being discussed, molecular characterization of these intermediate states might be helpful for wound healing research.

As mentioned before, there is a lack of specific knowledge on TGF-β and EMT in skin wound healing. A recently published study (2019) demonstrated the implication of both canonical and non-canonical TGF-β1 signaling for proper keratinocyte transdifferentiation and successful wound closure in the axolotl (*Ambystoma mexicanum*), since exposure to selective inhibitors for each pathway resulted in delayed re-epithelization [119]. Compared to humans, the axolotl shows accelerated wound closure also characterized by no scarring, both achieved through a molecular mechanism which involves TGF-β signaling [120]. In fact, several studies show how TGF-β signaling is necessary for tissue regeneration in other lower vertebrate models, including *Xenopus* and zebrafish [121,122]. This evidence provides unique hints on the role of TGF-β in keratinocyte transdifferentiation; however, the axolotl model itself is distant from human skin. At the molecular level, this is evidenced by the fact that only TGF-β1, but not TGF-β2 or TGF-β3, is detected in its regenerating tissues [123]. Moreover, the axolotl develops shorter TGF-β1 induction and scarce leukocyte infiltration during wound healing [124]. Interestingly, it has been suggested that the regenerative capacities shown by some lower vertebrates might be related to their neotenic potential (i.e., retaining typical traits of early stages of life) [125]. To that extent, it is well established that early-gestation human skin wounds repair quickly and without scar formation [126]. The mechanisms leading to this resolution of fetal wounds remain unknown. However, reviewed evidence on this issue points to TGF-β as the main factor involved, as in fetal skin, only TGF-β3 expression is found to be increased while TGF-β1 levels remain steady, in clear contrast with what is found in adults [127]. In that sense, studies performed on mice and rats provide evidence of a contrasting TGF-β1/TGF-β3 ratio between the skin and the oral mucosa during wound healing [128,129]. This observation is highly interesting, as oral mucosal wounds are indeed known to heal faster and with minimal scarring in comparison with skin wounds [130]. Interestingly, although evidence available in humans is restricted, a recent study analyzing oral scars appearing after oral tumor removal suggests a pattern of increased TGF-β1 detection [131]. Indeed, the treatment of adult skin wounds with exogenous TGF-β3 or, alternatively, with neutralizing antibodies for TGF-β1 and TGF-β2 has been suggested to reduce scar formation and improve aesthetics after healing [89,132]. Moreover, treatment of fetal wounds with TGF-β1 results in scarification [133]. Altogether, this evidence suggests that relative fractions of TGF-β isoforms, rather than absolute amounts, may direct the evolution of the wound through their impact on the regulation of gene expression and the release of cell mediators driving leukocyte recruitment, keratinocyte activation, or ECM deposition. 

## 10. Future Directions

Optimal TGF-β function seems essential for successful wound closure. The contribution of this cytokine to unbalanced signaling may play an important role in wounds becoming chronic. Yet, although this idea is potentially attractive, several reviews which analyzed therapies based on enhancing or interfering with TGF-β pathway showed how these strategies are historically unable to meet expectations [33,37,57]. To this effect, we should bear in mind that the integration of TGF-β signaling is highly dependent on the context and targeted cell. Thus, better understanding of TGF-β-specific roles in certain skin cell types might help to overcome the limitations found by these strategies. Consequently, discriminating of the sustained high TGF-β levels dominating the epidermal compartment from the reduced levels in the dermis of chronic wounds [30,81,82] poses an innovative paradigm worth studying. Yet, the complexity of the system involving direct and indirect activation mechanisms and parallel signaling by TGF-β isoforms and concurrent Smad-dependent and -independent pathways together with the crosstalk between cell types and compartments makes experimental design and interpretations of results very challenging. Nevertheless, some aspects can be anticipated as fundamental in tackling this issue. 

Firstly, regarding the characterization of TGF-β levels in chronic wounds, we believe that the collective evidence, exposed above, should be regarded as acceptable as it is sustained on consistent techniques performed through time by unrelated and different research groups. Yet, the total data from these studies account for 39 patients with chronic processes of different origins [30,81,82], maybe just enough to satisfy the minimums required for validating evidence. Not only increased levels but also the precise characterization of TGF-β levels in the current possible types of biological samples to be obtained from chronic wounds, also using up-to-date techniques, will benefit this understanding. In this regard, taking into account precise molecular aspects in the study design might by fundamental for obtaining useful knowledge for further stages. One valuable example would be considering if an antibody recognizes TGF-β by binding either all the isoforms, only a monomer, possibly dimers, perhaps simultaneously with a monomer and its LAP, or maybe the LAP alone. The precise and subtle differences in LAP sequences [5] within a matrix metalloproteinase (MMP)-congested scenario, such as a chronic wound [134], might be crucial in discriminating TGF-β expression from its activity and defined cell responses to TGF-β isoforms, an aspect already proven relevant for breast cancer physiology [135,136].

Here, we have gone into detail over the relationship between TGF-β and keratinocyte cell responses during wound healing, such as the effects of their activation and release of other cell mediators, the regulation of their proliferation and their involvement in the development of an EMT-like state necessary for cell migration. Thus, a second fundamental aspect might be constituted by the effects that continued exposure to TGF-β may have on keratinocyte responses. This might include known aspects such as the evolution of cell cycle responses, alterations of the activation cycle defined by changes in the expression of keratins, the modulation of gene expression or, last but not least, the induction of mechanisms related to EMT. In this regard, despite their great relevance for re-epithelization and closure, there are few studies addressing, in detail, specific mechanisms by which TGF-β promotes transdifferentiation of epidermis keratinocytes to acquire migratory potential during wound healing. Moreover, research might have overlooked the specific autocrine potential of TGF-β expressed by cells of the epidermis in the context of chronic wound healing. As mentioned earlier, expression of all TGF-β isoforms is induced when there is skin injury so that it slowly recovers as the wound healing completes [35]. In relation to this, keratinocyte differentiation during wound healing might involve sensitization to TGF-β signaling [97,98]. Taking all this into account, a possibility worth exploring is that long-term elevated TGF-β levels might contribute to the establishment of a closed signaling feedback in the context of stagnated re-epithelization.

Deeper knowledge of the paracrine effects of TGF-β produced in the skin could also be helpful. The crosstalk between epidermis and dermis has received limited attention, being focused mainly in inflammatory signals (reviewed in [1]). However, some evidence provides hints of the potential importance in chronic wounds of this relationship between keratinocytes, fibroblasts, and endothelial cells through TGF-β. On the one hand, fibroblasts isolated from chronic ulcers show non-functional TGF-β signaling, which fits the historical paradigm of decreased TGF-β activity in chronic wounds [92]. On the other hand, randomized controlled trials have shown that the application of negative pressure therapy (NPWT) to diabetic foot wounds promotes endothelial cell sprouting and granulation tissue functionality. Interestingly, these effects have been linked to fluctuations in TGF-β levels and, in fact, determining reduced TGF-β1 levels was useful for establishing an efficacy indicator of the treatment [137]. However, NPWT might not establish a definitive approach for chronic wounds, as re-epithelization sometimes fails to resume despite the application of this technique [138]. These are interesting observations, especially considering the opposite scenario regarding TGF-β levels found in the epidermis and the continued immune impulse which is typical of the dermis compartment. Thus far, exploring the origins and consequences of this fibroblast insensitivity—perhaps resistance—to TGF-β may provide better understanding of the events leading to wound chronification.

Another aspect would be better understanding the relationship between TGF-β and inflammation in the chronic wound context. Wound fluid samples obtained from chronic ulcers are known to be rich in pro-inflammatory cytokines such as TNFα and IL-1β. Also, isolation of TGF-β from these samples was reported some time ago [139]. The evidence obtained in transgenic and knockout mice overexpressing or lacking TGF-β1 in keratinocytes seems to support the interest of this point, as knockout skin is devoid of inflammation [43,44] while transgenic mice overexpressing TGF-β show neutrophil infiltrates both in the dermis and epidermis of non-ulcerated skin, with massive infiltration in ulcerated dermis [36,47]. In this regard and taking TGF-β pro-inflammatory properties into account, it may also be worth studying the ability of high and continued TGF-β levels to regulate the expression and release by keratinocytes of additional cytokines, affecting the behavior of immune cells in the epidermis and in the dermal compartment. 

## 11. Concluding Remarks

**Figure 1:**
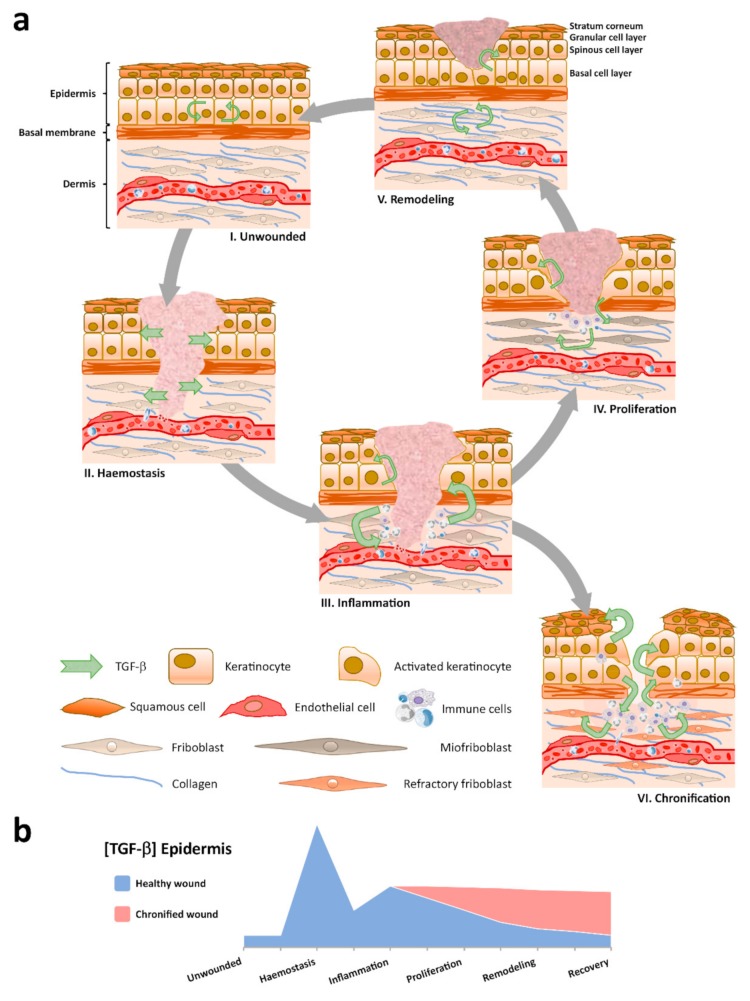
Spatial and temporal patterns for TGF-β evolve during wound healing. (**a**) Distinct TGF-β signatures appear through the skin tissue compartments: i) Low TGF-β levels are characteristic of the homeostatic epidermis in unwounded skin; ii) After injury, a clot is constituted and TGF-β, from degranulated platelets, diffuses into neighboring skin tissue compartments; iii) Immune cells infiltrate and contribute to the regional cytokine milieu; iv) Molecular crosstalk between skin tissue compartments coordinates wound contraction and re-epithelization; v) Paracrine signaling drives dermal extracellular matrix (ECM) replacement and epidermal homeostasis recovery to a low TGF-β concentration environment; vi) Persistent immune infiltrates and aberrant cell behaviors establish, including dermal fibroblast becoming refractory to TGF-β signaling and the development of reactive hyperkeratosis and parakeratosis on exacerbated TGF-β expression in the epidermis. (**b**) The quantity of TGF-β varies in the epidermis through the sequential stages of wound healing.

Chronic wounds are far from being a new pathological entity, however, their prevalence grows rapidly worldwide compared to pathologies associated with aging (e.g., diabetes, vascular insufficiency, restricted mobility, etc.). Innovative strategies are needed for their prevention and treatment. Nevertheless, there is a lack of profound understanding of the mechanisms involved despite research efforts. This situation limits progress because results of most clinical trials on this field are actually disappointing. Above all, this generates an increasing strain for health systems and society. Research on the extended implications of TGF-β signaling in chronic wounds, incorporating the paradigm developed in this paper (Figure 1), may represent an advantageous starting point in order to increase our current understanding. In this regard, despite being necessary, obtaining samples from patients represents a burdensome process most of the time, with patients often presenting with comorbidities. Consequently, finding and perfecting innovative research models based on human cells could constitute a convenient strategy for the study of specific cellular responses within the context of chronic wounds. Especially for keratinocytes, no definite model has been used to study continued responses to TGF-β. Trying to address this contingency, in another study published in this same issue of *Cells* [140], we offer original evidence on the utility of spontaneously immortalized human epidermal keratinocytes, HaCaT cells, a research model valued for the study of keratinocyte physiology. Our results disclose this model as a significant tool to understand cell behavior and molecular events leading to the impaired epithelization state found in chronic wounds.

## References

[1] Freedberg I.M., Tomic-Canic M., Komine M., Blumenberg M. (2001). Keratins and the Keratinocyte Activation Cycle. J. Investig. Dermatol..

[2] Castellanos G., Bernabé-García Á., Moraleda J.M., Nicolás F.J. (2017). Amniotic membrane application for the healing of chronic wounds and ulcers. Placenta.

[3] O’Kane S., Ferguson M.W. (1997). Transforming growth factor βs and wound healing. Int. J. Biochem. Cell Biol..

[4] Bielefeld K.A., Amini-Nik S., Alman B.A. (2013). Cutaneous wound healing: recruiting developmental pathways for regeneration. Cell Mol. Life Sci..

[5] Hinck A.P., Mueller T.D., Springer T.A. (2016). Structural Biology and Evolution of the TGF-β Family. Cold Spring Harb. Perspect. Biol..

[6] Chen Y.-G., Massagué J., Hata A., Lo R.S., Wotton D., Shi Y., Pavletich N. (1998). Determinants of specificity in TGF-β signal transduction. Genes Dev..

[7] Kiritsi D., Nyström A. (2018). The role of TGFβ in wound healing pathologies. Mech. Ageing Dev..

[8] Hinck A.P., Archer S.J., Qian S.W., Roberts A.B., Sporn M.B., Weatherbee J.A., Tsang M.L.-S., Lucas R., Zhang B.-L., Wenker J. (1996). Transforming Growth Factor β1: Three-Dimensional Structure in Solution and Comparison with the X-ray Structure of Transforming Growth Factor β2. Biochemistry.

[9] Mittl P.R.E., Priestle J.P., Cox D.A., McMaster G., Cerletti N., Grütter M.G. (1996). The crystal structure of TGF-β3 and comparison to TGF-β2: Implications for receptor binding. Protein Sci..

[10] Cheifetz S., Hernandez H., Laiho M., Dijke P.T., Iwata K.K., Massagué J. (1990). Distinct transforming growth factor-β (TGF-β) receptor subsets as determinants of cellular responsiveness to three TGF-β isoforms. J. Biol. Chem..

[11] Baardsnes J., Hinck C.S., Hinck A.P., O’Connor-McCourt M.D. (2009). TβR-II Discriminates the High- and Low-Affinity TGF-β Isoforms via Two Hydrogen-Bonded Ion Pairs. Biochemistry.

[12] Cheifetz S., Bassols A., Stanley K., Ohta M., Greenberger J., Massagué J. (1988). Heterodimeric transforming growth factor beta. Biological properties and interaction with three types of cell surface receptors. J. Biol. Chem..

[13] Ogawa Y., Schmidt D.K., Dasch J.R., Chang R.J., Glaser C.B. (1992). Purification and characterization of transforming growth factor-β2.3 and -β1.2 heterodimers from bovine bone. J. Biol. Chem..

[14] Annes J.P., Munger J.S., Rifkin D.B. (2003). Making sense of latent TGFβ activation. J. Cell Sci..

[15] Shi M., Zhu J., Wang R., Chen X., Mi L., Walz T., Springer T.A. (2011). Latent TGF-β structure and activation. Nature.

[16] Soo C., Hu F.-Y., Zhang X., Wang Y., Beanes S.R., Lorenz H.P., Hedrick M.H., MacKool R.J., Plaas A., Kim S.-J. (2000). Differential Expression of Fibromodulin, a Transforming Growth Factor-β Modulator, in Fetal Skin Development and Scarless Repair. Am. J. Pathol..

[17] Robertson I.B., Horiguchi M., Zilberberg L., Dabovic B., Hadjiolova K., Rifkin D.B. (2015). Latent TGF-β-binding proteins. Matrix. Biol..

[18] Nickel J., Ten Dijke P., Mueller T.D. (2018). TGF-β family co-receptor function and signaling. Acta Biochim. Biophys. Sin..

[19] Bruce D.L., Sapkota G.P. (2012). Phosphatases in SMAD regulation. FEBS Lett..

[20] García-Vizcaíno E.M., Liarte S., Alonso-Romero J.L., Nicolás F.J. (2017). Sirt1 interaction with active Smad2 modulates transforming growth factor-β regulated transcription. Cell Commun. Signal..

[21] Feng X.-H., Derynck R. (2005). Specificity and versatility in TGF-β signaling through Smads. Annu. Rev. Cell Dev. Biol..

[22] Moustakas A., Heldin C.H. (2005). Non-Smad TGF-β signals. J. Cell Sci..

[23] Mu Y., Gudey S.K., Landstrom M. (2012). Non-Smad signaling pathways. Cell Tissue Res..

[24] Weiss A., Attisano L. (2013). The TGFβ superfamily signaling pathway. Wiley Interdiscip. Rev. Dev. Biol..

[25] Attisano L., Wrana J.L. (2013). Signal integration in TGF-β, WNT, and Hippo pathways. F1000Prime Rep..

[26] Luo K. (2017). Signaling Cross Talk between TGF-β/Smad and Other Signaling Pathways. Cold Spring Harb. Perspect. Biol..

[27] Bai J., Xi Q. (2018). Crosstalk between TGF-β signaling and epigenome. Acta Biochim. Biophys. Sin..

[28] Suzuki H.I. (2018). MicroRNA Control of TGF-β Signaling. Int. J. Mol. Sci..

[29] Kane C.J.M., Hebda P.A., Mansbridge J.N., Hanawalt P.C. (1991). Direct evidence for spatial and temporal regulation of transforming growth factor β1 expression during cutaneous wound healing. J. Cell. Physiol..

[30] Schmid P., Cox D., Bilbe G., McMaster G., Morrison C., Stähelin H., Lüscher N., Seiler W. (1993). TGF-βS and TGF-β type II receptor in human epidermis: Differential expression in acute and chronic skin wounds. J. Pathol..

[31] Jiang C.K., Tomić-Canić M., Lucas D.J., Simon M., Blumenberg M. (1995). TGF beta promotes the basal phenotype of epidermal keratinocytes: transcriptional induction of K#5 and K#14 keratin genes. Growth Factors.

[32] Ramírez H., Patel S.B., Pastar I. (2014). The Role of TGFβ Signaling in Wound Epithelialization. Adv. Wound Care.

[33] Gilbert R.W., Vickaryous M.K., Viloria-Petit A.M. (2016). Signalling by Transforming Growth Factor Beta Isoforms in Wound Healing and Tissue Regeneration. J. Dev. Biol..

[34] Frank S., Madlener M., Werner S. (1996). Transforming Growth Factors 1, 2, and 3 and Their Receptors Are Differentially Regulated during Normal and Impaired Wound Healing. J. Biol. Chem..

[35] Yang L., Qiu C.X., Ludlow A., Ferguson M.W., Brunner G. (1999). Active transforming growth factor-beta in wound repair: determination using a new assay. Am. J. Pathol..

[36] Wang X.-J., Han G., Owens P., Siddiqui Y., Li A.G. (2006). Role of TGFβ-mediated inflammation in cutaneous wound healing. J. Investig. Dermatol. Symp. Proc..

[37] Barrientos S., Stojadinovic O., Golinko M.S., Brem H., Tomic-Canic M. (2008). Perspective Article: Growth factors and cytokines in wound healing. Wound Repair Regen..

[38] Valluru M., Staton C.A., Reed M.W.R., Brown N.J. (2011). Transforming Growth Factor-β and Endoglin Signaling Orchestrate Wound Healing. Front. Physiol..

[39] Morikawa M., Derynck R., Miyazono K. (2016). TGF-β and the TGF-β Family: Context-Dependent Roles in Cell and Tissue Physiology. Cold Spring Harb. Perspect. Biol..

[40] Mauviel A. (2009). Transforming growth factor-beta signaling in skin: stromal to epithelial cross-talk. J. Investig. Dermatol..

[41] Le Poole B. (1999). Keratinocytes suppress transforming growth factor-β1 expression by fibroblasts in cultured skin substitutes. Br. J. Dermatol..

[42] Li A.G., Lu S.-L., Han G., Hoot K.E., Wang X.-J. (2006). Role of TGFβ in skin inflammation and carcinogenesis. Mol. Carcinog..

[43] Shull M.M., Ormsby I., Kier A.B., Pawlowski S., Diebold R.J., Yin M., Allen R., Sidman C., Proetzel G., Calvin D. (1992). Targeted disruption of the mouse transforming growth factor-beta 1 gene results in multifocal inflammatory disease. Nature.

[44] Kulkarni A.B., Huh C.G., Becker D., Geiser A., Lyght M., Flanders K.C., Roberts A.B., Sporn M.B., Ward J.M., Karlsson S. (1993). Transforming growth factor beta 1 null mutation in mice causes excessive inflammatory response and early death. Proc. Natl. Acad. Sci. USA.

[45] Das L., Levine A.D. (2008). TGF-β Inhibits IL-2 Production and Promotes Cell Cycle Arrest in TCR-Activated Effector/Memory T Cells in the Presence of Sustained TCR Signal Transduction. J. Immunol..

[46] Gutcher I., Donkor M.K., Ma Q., Rudensky A.Y., Flavell R.A., Li M.O. (2011). Autocrine transforming growth factor-β1 promotes in vivo Th17 cell differentiation. Immunity.

[47] Han G., Li F., Singh T.P., Wolf P., Wang X.-J. (2012). The Pro-inflammatory Role of TGFβ1: A Paradox?. Int. J. Biol. Sci..

[48] Liu X., Alexander V., Vijayachandra K., Bhogte E., Diamond I., Glick A. (2001). Conditional epidermal expression of TGFβ1 blocks neonatal lethality but causes a reversible hyperplasia and alopecia. Proc. Natl. Acad. Sci. USA.

[49] Chan T., Ghahary A., Demare J., Yang L., Iwashina T., Scott P.G., Tredget E.E. (2002). Development, characterization, and wound healing of the keratin 14 promoted transforming growth factor-beta1 transgenic mouse. Wound Repair Regen..

[50] Li A.G., Wang D., Feng X.-H., Wang X.-J. (2004). Latent TGFβ1 overexpression in keratinocytes results in a severe psoriasis-like skin disorder. EMBO J..

[51] Ashcroft G.S., Yang X., Glick A.B., Weinstein M., Letterio J.J., Mizel D.E., Anzano M., Greenwell-Wild T., Wahl S.M., Deng C. (1999). Mice lacking Smad3 show accelerated wound healing and an impaired local inflammatory response. Nature.

[52] Yang X., Letterio J.J., Lechleider R.J., Chen L., Hayman R., Gu H., Roberts A.B., Deng C. (1999). Targeted disruption of SMAD3 results in impaired mucosal immunity and diminished T cell responsiveness to TGF-β. EMBO J..

[53] Han G., Williams C.A., Salter K., Garl P.J., Li A.G., Wang X.J. (2010). A role for TGFβ signaling in the pathogenesis of psoriasis. J. Invest. Dermatol..

[54] Sferra R., Fargnoli M.C., Corbelli E., Pellegrini C., Peris K., Gaudio E., Vetuschi A. (2014). Immunopathogenesis of psoriasis: a possible role of TGFβ/Smads pathway. Ital. J. Anat. Embryol..

[55] Zhang Y., Meng X.-M., Huang X.-R., Wang X.-J., Yang L., Lan H.Y. (2014). Transforming growth factor-β1 mediates psoriasis-like lesions via a Smad3-dependent mechanism in mice. Clin. Exp. Pharmacol. Physiol..

[56] Pastar I., Stojadinovic O., Yin N.C., Ramírez H., Nusbaum A.G., Sawaya A., Patel S.B., Khalid L., Isseroff R.R., Tomic-Canic M. (2014). Epithelialization in Wound Healing: A Comprehensive Review. Adv. Wound Care.

[57] Finnson K.W., McLean S., Di Guglielmo G.M., Philip A. (2013). Dynamics of Transforming Growth Factor Beta Signaling in Wound Healing and Scarring. Adv. Wound Care.

[58] Mustoe T., Pierce G., Thomason A., Gramates P., Sporn M., Deuel T. (1987). Accelerated healing of incisional wounds in rats induced by transforming growth factor-beta. Science.

[59] Pierce G.F., Mustoe T.A., Lingelbach J., Masakowski V.R., Gramates P., Deuel T.F. (1989). Transforming growth factor beta reverses the glucocorticoid-induced wound-healing deficit in rats: possible regulation in macrophages by platelet-derived growth factor. Proc. Natl. Acad. Sci. USA.

[60] Salomon G.D., Kasid A., Bernstein E., Buresh C., Director E., A Norton J. (1990). Gene expression in normal and doxorubicin-impaired wounds: importance of transforming growth factor-beta. Surgery.

[61] Beck L.S., DeGuzman L., Lee W.P., Xu Y., A McFatridge L., Amento E.P. (1991). TGF-beta 1 accelerates wound healing: reversal of steroid-impaired healing in rats and rabbits. Growth Factors.

[62] Beck L.S., DeGuzman L., Lee W.P., Xu Y., Siegel M.W., Amento E.P. (1993). One systemic administration of transforming growth factor-beta 1 reverses age- or glucocorticoid-impaired wound healing. J. Clin. Investig..

[63] Crowe M.J., Doetschman T., Greenhalgh D.G. (2000). Delayed Wound Healing in Immunodeficient TGF-β1 Knockout Mice. J. Investig. Dermatol..

[64] Lee P.-Y., Chesnoy S., Huang L. (2004). Electroporatic Delivery of TGF-β1 Gene Works Synergistically with Electric Therapy to Enhance Diabetic Wound Healing in db/db Mice. J. Investig. Dermatol..

[65] Tredget E.B., Demare J., Chandran G., Tredget E.E., Yang L., Ghahary A. (2005). Transforming growth factor-β and its effect on reepithelialization of partial-thickness ear wounds in transgenic mice. Wound Repair Regen..

[66] Lu L., Saulis A.S., Liu W.R., Roy N.K., Chao J.D., Ledbetter S., Mustoe T.A. (2005). The Temporal Effects of Anti-TGF-β1, 2, and 3 Monoclonal Antibody on Wound Healing and Hypertrophic Scar Formation. J. Am. Coll. Surg..

[67] El Gazaerly H., Elbardisey D.M., Eltokhy H.M. (2013). Effect of Transforming Growth Factor Beta 1 on Wound Healing in Induced Diabetic Rats. Int. J. Heal. Sci..

[68] Wu L., Xia Y.P., Roth S.I., Gruskin E., Mustoe T.A. (1999). Transforming growth factor-beta1 fails to stimulate wound healing and impairs its signal transduction in an aged ischemic ulcer model: importance of oxygen and age. Am. J. Pathol..

[69] Koch R.M., Roche N.S., Parks W.T., Ashcroft G.S., Letterio J.J., Roberts A.B. (2000). Incisional wound healing in transforming growth factor-β1 null mice. Wound Repair Regen..

[70] Grose R., Werner S. (2004). Wound-Healing Studies in Transgenic and Knockout Mice. Mol. Biotechnol..

[71] Robson M.C., Phillip L.G., Cooper D.M., Lyle W.G., Robson L.E., Odom L., Hill D.P., Hanham A.F., Ksander G.A. (1995). Safety and effect of transforming growth factor-β2 for treatment of venous stasis ulcers. Wound Repair Regen..

[72] Bennett S.P., Griffiths G.D., Schor A.M., Leese G.P., Schor S.L. (2003). Growth factors in the treatment of diabetic foot ulcers. BJS.

[73] Finnson K.W., Arany P.R., Philip A. (2013). Transforming Growth Factor Beta Signaling in Cutaneous Wound Healing: Lessons Learned from Animal Studies. Adv. Wound Care.

[74] Rittie L., Sachs D.L., Orringer J.S., Voorhees J.J., Fisher G.J. (2013). Eccrine sweat glands are major contributors to reepithelialization of human wounds. Am. J. Pathol..

[75] Martínez M.-L., Escario E., Poblet E., Sánchez D., Buchón F.-F., Izeta A., Jimenez F. (2016). Hair follicle–containing punch grafts accelerate chronic ulcer healing: A randomized controlled trial. J. Am. Acad. Dermatol..

[76] Poblet E., Jimenez F., Escario-Travesedo E., Hardman J.A., Hernandez-Hernandez I., Agudo-Mena J.L., Cabrera-Galvan J.J., Nicu C., Paus R. (2018). Eccrine sweat glands associate with the human hair follicle within a defined compartment of dermal white adipose tissue. Br. J. Dermatol..

[77] Hibino T., Nishiyama T. (2004). Role of TGF-β2 in the human hair cycle. J. Dermatol. Sci..

[78] Jamora C., Lee P., Kocieniewski P., Azhar M., Hosokawa R., Chai Y., Fuchs E. (2005). A signaling pathway involving TGF-β2 and snail in hair follicle morphogenesis. PLoS Biol..

[79] Inoue K., Aoi N., Yamauchi Y., Sato T., Suga H., Eto H., Kato H., Tabata Y., Yoshimura K. (2009). TGF-β2 is specifically expressed in human dermal papilla cells and modulates hair folliculogenesis. J. Cell. Mol. Med..

[80] Oshimori N., Fuchs E. (2012). Paracrine TGF-β signaling counterbalances BMP-mediated repression in hair follicle stem cell activation. Cell Stem Cell.

[81] Cowin A.J., Hatzirodos N., Holding C.A., Dunaiski V., Rayner T.E., Harries R.H., Fitridge R., Cooter R.D., Schultz G.S., Belford D.A. (2001). Effect of Healing on the Expression of Transforming Growth Factor βs and their Receptors in Chronic Venous Leg Ulcers. J. Investig. Dermatol..

[82] Jude E.B., Blakytny R., Bulmer J., Boulton A.J.M., Ferguson M.W.J. (2002). Transforming growth factor-beta 1, 2, 3 and receptor type I and II in diabetic foot ulcers. Diabet. Med..

[83] Faler B.J., A Macsata R., Plummer D., Mishra L., Sidawy A.N. (2006). Transforming growth factor-beta and wound healing. Perspect. Vasc. Surg. Endovasc. Ther..

[84] Varga J., Rosenbloom J., A Jimenez S. (1987). Transforming growth factor β (TGFβ) causes a persistent increase in steady-state amounts of type I and type III collagen and fibronectin mRNAs in normal human dermal fibroblasts. Biochem. J..

[85] Hocevar B.A., Brown T.L., Howe P.H. (1999). TGF-beta induces fibronectin synthesis through a c-Jun N-terminal kinase-dependent, Smad4-independent pathway. EMBO J..

[86] Cordeiro M.F., Bhattacharya S.S., Schultz G.S., Khaw P.T. (2000). TGF-β1, -β2, and -β3 in vitro: biphasic effects on Tenon’s fibroblast contraction, proliferation, and migration. Invest. Ophthalmol. Vis. Sci..

[87] Desmoulière A. (1993). Transforming growth factor-beta 1 induces alpha-smooth muscle actin expression in granulation tissue myofibroblasts and in quiescent and growing cultured fibroblasts. J. Cell Biol..

[88] Martinez-Ferrer M., Afshar-Sherif A.-R., Uwamariya C., De Crombrugghe B., Davidson J.M., Bhowmick N.A. (2010). Dermal Transforming Growth Factor-β Responsiveness Mediates Wound Contraction and Epithelial Closure. Am. J. Pathol..

[89] Shah M., Foreman D.M., Ferguson M.W. (1995). Neutralisation of TGF-beta 1 and TGF-beta 2 or exogenous addition of TGF-beta 3 to cutaneous rat wounds reduces scarring. J. Cell Sci..

[90] Murata H., Zhou L., Ochoa S., Hasan A., Badiavas E., Falanga V. (1997). TGF-β3 Stimulates and Regulates Collagen Synthesis Through TGF-β1-Dependent and Independent Mechanisms. J. Investig. Dermatol..

[91] Chalmers R.L. (2011). The evidence for the role of transforming growth factor-beta in the formation of abnormal scarring. Int. Wound J..

[92] Kim B.-C., Kim H.T., Park S.H., Cha J.-S., Yufit T., Kim S.-J., Falanga V. (2003). Fibroblasts from chronic wounds show altered TGF-β-signaling and decreased TGF-β Type II Receptor expression. J. Cell. Physiol..

[93] Jeong H.W., Kim I.S. (2004). TGF-beta1 enhances betaig-h3-mediated keratinocyte cell migration through the α3β1 integrin and PI3K. J. Cell Biochem..

[94] Heldin C.-H., Landström M., Moustakas A. (2009). Mechanism of TGF-β signaling to growth arrest, apoptosis, and epithelial–mesenchymal transition. Curr. Opin. Cell Biol..

[95] Davies M., Robinson M., Smith E., Huntley S., Prime S., Paterson I. (2005). Induction of an epithelial to mesenchymal transition in human immortal and malignant keratinocytes by TGF-β1 involves MAPK, Smad and AP-1 signalling pathways. J. Cell. Biochem..

[96] Cammareri P., Rose A.M., Vincent D.F., Wang J., Nagano A., Libertini S., Ridgway R.A., Athineos D., Coates P.J., McHugh A. (2016). Inactivation of TGFβ receptors in stem cells drives cutaneous squamous cell carcinoma. Nat. Commun..

[97] Cho H.-R., Hong S.-B., Kim Y.I., Lee J.-W., Kim N.-I. (2004). Differential Expression of TGF-β Isoforms During Differentiation of HaCaT Human Keratinocyte Cells: Implication for the Separate Role in Epidermal Differentiation. J. Korean Med Sci..

[98] Duan D., Derynck R. (2019). Transforming growth factor-β (TGF-β)-induced up-regulation of TGF-β receptors at the cell surface amplifies the TGF-β response. J. Biol. Chem..

[99] Park S., Gonzalez D.G., Guirao B., Boucher J.D., Cockburn K., Marsh E.D., Mesa K.R., Brown S., Rompolas P., Haberman A.M. (2017). Tissue-scale coordination of cellular behaviour promotes epidermal wound repair in live mice. Nature.

[100] Aragona M., Dekoninck S., Rulands S., Lenglez S., Mascré G., Simons B.D., Blanpain C. (2017). Defining stem cell dynamics and migration during wound healing in mouse skin epidermis. Nat. Commun..

[101] Xie Y., Gao K., Häkkinen L., Larjava H.S. (2009). Mice lacking β6 integrin in skin show accelerated wound repair in dexamethasone impaired wound healing model. Wound Repair Regen..

[102] Sellheyer K., Bickenbach J.R., Rothnagel J.A., Bundman D., Longley M.A., Krieg T., Roche N.S., Roberts A.B., Roop D.R. (1993). Inhibition of skin development by overexpression of transforming growth factor beta 1 in the epidermis of transgenic mice. Proc. Natl. Acad. Sci. USA.

[103] Yang L., Chan T., Demare J., Iwashina T., Ghahary A., Scott P.G., Tredget E.E. (2001). Healing of Burn Wounds in Transgenic Mice Overexpressing Transforming Growth Factor-β1 in the Epidermis. Am. J. Pathol..

[104] Brown R.L., Ormsby I., Doetschman T.C., Greenhalgh D.G. (1995). Wound healing in the transforming growth factor-beta1-deficient mouse. Wound Repair Regen..

[105] Kalluri R., Weinberg R.A. (2009). The basics of epithelial-mesenchymal transition. J. Clin. Investig..

[106] Xu J., Lamouille S., Derynck R. (2009). TGF-β-induced epithelial to mesenchymal transition. Cell Res..

[107] Hao Y., Baker D., Dijke P.T. (2019). TGF-β-Mediated Epithelial-Mesenchymal Transition and Cancer Metastasis. Int. J. Mol. Sci..

[108] Wendt M.K., Allington T.M., Schiemann W.P. (2009). Mechanisms of the epithelial–mesenchymal transition by TGF-β. Futur. Oncol..

[109] Heldin C.-H., Vanlandewijck M., Moustakas A. (2012). Regulation of EMT by TGFβ in cancer. FEBS Lett..

[110] Wendt M.K., Tian M., Schiemann W.P. (2012). Deconstructing the mechanisms and consequences of TGF-beta-induced EMT during cancer progression. Cell Tissue Res..

[111] Naber H.P., Drabsch Y., Snaar-Jagalska B.E., Dijke P.T., Van Laar T. (2013). Snail and Slug, key regulators of TGF-β-induced EMT, are sufficient for the induction of single-cell invasion. Biochem. Biophys. Res. Commun..

[112] Bolós V., Peinado H., Pérez-Moreno M.A., Fraga M.F., Esteller M., Cano A. (2003). The transcription factor Slug represses E-cadherin expression and induces epithelial to mesenchymal transitions: a comparison with Snail and E47 repressors. J. Cell Sci..

[113] Gui T., Sun Y., Shimokado A., Muragaki Y. (2012). The Roles of Mitogen-Activated Protein Kinase Pathways in TGF-β-Induced Epithelial-Mesenchymal Transition. J. Signal Transduct..

[114] Haensel D., Dai X. (2018). Epithelial-to-mesenchymal transition in cutaneous wound healing: Where we are and where we are heading. Dev. Dyn..

[115] Rousselle P., Braye F., Dayan G. (2018). Re-epithelialization of adult skin wounds: Cellular mechanisms and therapeutic strategies. Adv. Drug Deliv. Rev..

[116] Weber C.E., Li N.Y., Wai P.Y., Kuo P.C. (2012). Epithelial-mesenchymal transition, TGF-β, and osteopontin in wound healing and tissue remodeling after injury. J. Burn. Care Res..

[117] Nieto M.A., Huang R.Y.-J., Jackson R.A., Thiery J.P. (2016). EMT: 2016. Cell.

[118] Sha Y., Haensel D., Gutierrez G., Du H., Dai X., Nie Q. (2019). Intermediate cell states in epithelial-to-mesenchymal transition. Phys. Biol..

[119] Sader F., Denis J.-F., Laref H., Roy S. (2019). Epithelial to mesenchymal transition is mediated by both TGF-β canonical and non-canonical signaling during axolotl limb regeneration. Sci. Rep..

[120] Lévesque M., Gatien S., Finnson K., Desmeules S., Villiard É., Pilote M., Philip A., Roy S. (2007). Transforming Growth Factor: β Signaling Is Essential for Limb Regeneration in Axolotls. PLoS ONE.

[121] Ho D.M., Whitman M. (2008). TGF-beta signaling is required for multiple processes during Xenopus tail regeneration. Dev. Biol..

[122] Chablais F., Jazwinska A. (2012). The regenerative capacity of the zebrafish heart is dependent on TGFβ signaling. Development.

[123] Denis J.-F., Levesque M., Tran S.D., Camarda A.-J., Roy S. (2013). Axolotl as a Model to Study Scarless Wound Healing in Vertebrates: Role of the Transforming Growth Factor Beta Signaling Pathway. Adv. Wound Care.

[124] Levesque M., Villiard É., Roy S. (2010). Skin wound healing in axolotls: A scarless process. J. Exp. Zool. Part B Mol. Dev. Evol..

[125] Godwin J.W., Rosenthal N. (2014). Scar-free wound healing and regeneration in amphibians: Immunological influences on regenerative success. Differentiation.

[126] Larson B.J., Longaker M.T., Lorenz H.P. (2010). Scarless fetal wound healing: a basic science review. Plast. Reconstr. Surg..

[127] Ferguson M.W.J., O’Kane S. (2004). Scar-free healing: from embryonic mechanisms to adult therapeutic intervention. Philos. Trans. R. Soc. B Biol. Sci..

[128] Schrementi M.E., Ferreira A.M., Zender C., DiPietro L.A. (2008). Site-specific production of TGF-? in oral mucosal and cutaneous wounds. Wound Repair Regen..

[129] Zhao J.L.J., Liu J.Z.J. (2015). The Expression Level of TGF-β1, TGF-β3 and VEGF in Transplanted Oral Mucosal and Cutaneous Wounds. Clin. Microbiol..

[130] Iglesias-Bartolome R., Uchiyama A., Molinolo A.A., Abusleme L., Brooks S.R., Callejas-Valera J.L., Edwards D., Doci C., Asselin-Labat M.-L., Onaitis M.W. (2018). Transcriptional signature primes human oral mucosa for rapid wound healing. Sci. Transl. Med..

[131] Bucur M., Dinca O., Vladan C., Popp C., Nichita L., Cioplea M., Stînga P., Mustatea P., Zurac S., Ionescu E. (2018). Variation in Expression of Inflammation-Related Signaling Molecules with Profibrotic and Antifibrotic Effects in Cutaneous and Oral Mucosa Scars. J. Immunol. Res..

[132] So K., McGrouther D.A., Bush J.A., Durani P., Taylor L., Skotny G., Mason T., Metcalfe A., Oʼkane S., Ferguson M.W.J. (2011). Avotermin for Scar Improvement following Scar Revision Surgery: A Randomized, Double-Blind, Within-Patient, Placebo-Controlled, Phase II Clinical Trial. Plast. Reconstr. Surg..

[133] Sullivan K.M., Lorenz H., Meuli M., Lin R.Y., Adzick N. (1995). A model of scarless human fetal wound repair is deficient in transforming growth factor beta. J. Pediatr. Surg..

[134] Caley M.P., Martins V.L., O’Toole E.A. (2015). Metalloproteinases and Wound Healing. Adv. Wound Care.

[135] Barcellos-Hoff M.H., Ehrhart E.J., Kalia M., Jirtle R., Flanders K., Tsang M.L. (1995). Immunohistochemical detection of active transforming growth factor-beta in situ using engineered tissue. Am. J. Pathol..

[136] Ewan K.B., Shyamala G., Ravani S.A., Tang Y., Akhurst R., Wakefield L., Barcellos-Hoff M.H. (2002). Latent transforming growth factor-beta activation in mammary gland: regulation by ovarian hormones affects ductal and alveolar proliferation. Am. J. Pathol..

[137] Yang S.L., Zhu L.Y., Han R., Sun L.L., Dou J.T. (2017). Effect of Negative Pressure Wound Therapy on Cellular Fibronectin and Transforming Growth Factor-β1 Expression in Diabetic Foot Wounds. Foot Ankle Int..

[138] Castellanos G., Bernabé-García Á., Insausti C.G., Piñero A., Moraleda J.M., Nicolás F.J. (2016). The Use of Amniotic Membrane in the Management of Complex Chronic Wounds. Wound Healing.

[139] Harris I.R., Yee K.C., Walters C.E., Cunliffe W.J., Kearney J.N., Wood E.J., Ingham E. (1995). Cytokine and protease levels in healing and non-healing chronic venous leg ulcers. Exp. Dermatol..

[140] Liarte S., Bernabé-García Á., Nicolás F.J. (2020). Human Skin Keratinocytes on Sustained TGF-β Stimulation Reveal Partial EMT Features and Weaken Growth Arrest Responses. Cells.

